# Boosting Photo-to-Thermal Conversion and 1-Nitronaphthalene Reduction in Fe-MOF via Incorporating Carbon Nanotubes Heat-Storage Cocatalyst

**DOI:** 10.3390/nano16130817

**Published:** 2026-07-02

**Authors:** Ying-Cong Wei, Zhuang Miao, Zhipeng Xie, Xiong-Feng Ma

**Affiliations:** 1 College of Engineering, Xi’an International University, Xi’an 710077, China; xaiu24312@xaiu.edu.cn (Y.-C.W.); miaozhuang@xaiu.edu.cn (Z.M.); 2 Department of Chemistry, The Chinese University of Hong Kong, New Territories, Hong Kong 999077, China

**Keywords:** metal–organic frameworks, photothermal catalysis, nitronaphthalene reduction, carbon nanotubes

## Abstract

The development of efficient and sustainable photothermal catalytic systems is pivotal for modern organic transformations. Herein, we report the rational design and solvothermal synthesis of NH_2_-MIL-101(Fe) metal–organic frameworks (NM-101) integrated with carbon nanotubes (CNTs) for the photothermal reduction in nitronaphthalene. The optimized NM-101/75C composites exhibit exceptional catalytic activity and high selectivity under NIR light irradiation, delivering a high yield of 84.4% within 1 h, which significantly outperforms its individual components. Systematic control experiments and detailed spectroscopic investigations reveal a powerful synergistic effect at the MOF-CNT interface, where the CNTs play a dual role in augmenting light harvesting and facilitating charge carrier separation. Furthermore, the high photothermal conversion efficiency of the composite enables rapid reaction kinetics. This work provides a robust and scalable strategy for constructing high-performance photothermal catalysts, offering critical insights into the interfacial engineering of MOF-based materials for industrial chemical manufacturing.

## 1. Introduction

As a vital building block for azo dyes, agrochemicals, and high-performance polymers, 1-naphthylamine is indispensable to the fine chemical industry [1,2,3,4,5,6,7]. Industrial production of 1-naphthylamine traditionally requires the catalytic hydrogenation of 1-nitronaphthalene under harsh temperature and pressure [8]. However, these conventional thermal routes are often hindered by excessive energy consumption, reliance on costly noble-metal catalysts (e.g., Pd, Pt), and suboptimal selectivity [9].

Solar-driven photocatalysis offers a sustainable alternative to conventional thermal processes [10,11,12,13,14]. Nevertheless, the efficiency of pristine photocatalysts is frequently limited by rapid charge recombination and poor utilization of the solar spectrum, particularly in the infrared region [15,16,17,18]. In this context, photothermal catalysis has garnered significant attention as it synergistically harnesses energetic electronic excitation and localized thermal effects to lower reaction barriers and accelerate kinetic rates [19,20,21,22]. Consequently, the development of earth-abundant, non-noble-metal catalysts that integrate broad-spectrum absorption with efficient charge separation and high photothermal conversion efficiency remains a paramount challenge.

Metal–organic frameworks (MOFs), specifically the iron-based MIL-101 series, have demonstrated remarkable potential in heterogeneous catalysis owing to their ultrahigh surface areas, atomically dispersed active sites, and robust chemical stability [23,24,25,26,27,28,29,30,31,32,33,34,35]. By incorporating amino functional groups into the ligands, NH_2_-MIL-101(Fe) exhibits significantly enhanced visible-light harvesting capabilities [36]. However, its low intrinsic conductivity hinders the efficient migration of photogenerated carriers. Although noble metals such as Pt, Au, and Pd are highly effective cocatalysts for improving photocatalytic performance, their widespread application is hindered by high cost, limited availability, and long-term stability concerns. Carbon nanotubes (CNTs) exhibit an exceptional strength-to-weight ratio, diamond-like thermal conductivity, and robust chemical stability against harsh corrosive media [37,38,39,40]. Crucially, their electronic properties are highly tunable, displaying either metallic or semiconducting behaviors dictated by their structural chirality, which positions them as ideal building blocks for advanced functional composites and nanoelectronics [41,42,43,44,45,46]. The integration of CNTs into the MOF matrix can establish high-speed pathways for electron transport. More crucially, the exceptional photothermal effect of CNTs enables the conversion of near-infrared light into localized thermal energy, which, together with the photoactive centers of the MOF, creates a synergistic micro-environment that facilitates the reduction in nitro groups.

In this work, we report the rational design and synthesis of NH_2_-MIL-101(Fe)/CNTs composites (NM-101/C) for the efficient photothermal reduction in nitronaphthalene. The optimized NM-101/75C catalysts exhibit exceptional catalytic activity and high selectivity, achieving an impressive yield of 84.4% within only 1 h of irradiation under mild conditions. Through systematic control experiments and comprehensive spectroscopic analysis, we delineate the dual role of CNTs in facilitating charge carrier separation and augmenting light-to-heat conversion. This synergistic interplay at the MOF–carbon interface provides critical insights into the photothermal enhancement mechanism. Our work establishes a versatile and cost-effective strategy for engineering high-performance photothermal catalytic systems tailored for sustainable organic transformations.

## 2. Materials and Methods

### 2.1. Synthesis of NM-101

NM-101 was synthesized via a one-step solvothermal method. Typically, 15 mL of *N*,*N*-dimethylformamide (DMF) was employed to dissolve 2-aminoterephthalic acid (1.24 mmol) to form Solution 1. In parallel, FeCl_3_·6H_2_O (2.50 mmol) was dissolved in 15 mL of DMF to obtain Solution 2. Solution 1 was added dropwise into Solution 2 under constant stirring, and the resulting mixture was ultrasonicated for 30 min. The resulting mixture was then sealed inside a 100 mL Teflon-lined stainless-steel autoclave and maintained at 110 °C for a duration of 24 h. After cooling to ambient temperature naturally, the reddish-brown precipitate was collected by centrifugation (10,000 rpm, 6 min) and washed with DMF and ethanol. Finally, the product was dried at 80 °C under vacuum to yield NM-101 as a reddish-brown powder.

### 2.2. Synthesis of NM-101/XC Composites

The NM-101/XC (X = 35, 55, 75, and 95) photocatalysts were prepared via a facial solvothermal method. In a typical procedure for NM-101/75C, FeCl_3_·6H_2_O (0.27 g, 1.0 mmol) and 2-aminoterephthalic acid (NH2-BDC, 0.09 g, 0.5 mmol) were dispersed in 50 mL of *N*,*N*-dimethylformamide (DMF) under stirring. Subsequently, 75 mg of carbon nanotube were added. Then, the mixture was stirred for 1 h to obtain a homogeneous precursor suspension. The suspension was transferred to a Teflon-lined stainless-steel autoclave and maintained at 110 °C for 24 h. After cooling to ambient temperature naturally, the precipitate was isolated by centrifugation, washed thoroughly with DMF and ethanol, and dried at 80 °C overnight. Other composites, including NM-101/35C, NM-101/55C, and NM-101/95C, were synthesized following the same protocol by adjusting the initial mass of C fibers to 35 mg, 55 mg, and 95 mg, respectively.

### 2.3. Photocatalytic Reduction of 1-Nitronaphthalene

In a typical procedure, 1-nitronaphthalene (0.5 mmol), the as-prepared catalyst (10 mg), hydrazine monohydrate (85 wt%), and 1 mL of organic solvent were charged into a glass reactor. The reactor was hermetically sealed and stirred at ambient temperature under irradiation from a LED. After 1 h, the gaseous products in the headspace were analyzed using an online gas chromatograph (GC-7900, Techcomp, Shanghai, China) equipped with an argon carrier gas. The resulting amine products in the liquid phase were identified and quantified using a gas chromatograph equipped with an FID.

## 3. Results and Discussion

### 3.1. Characterizations of the Samples

**Figure 1:**
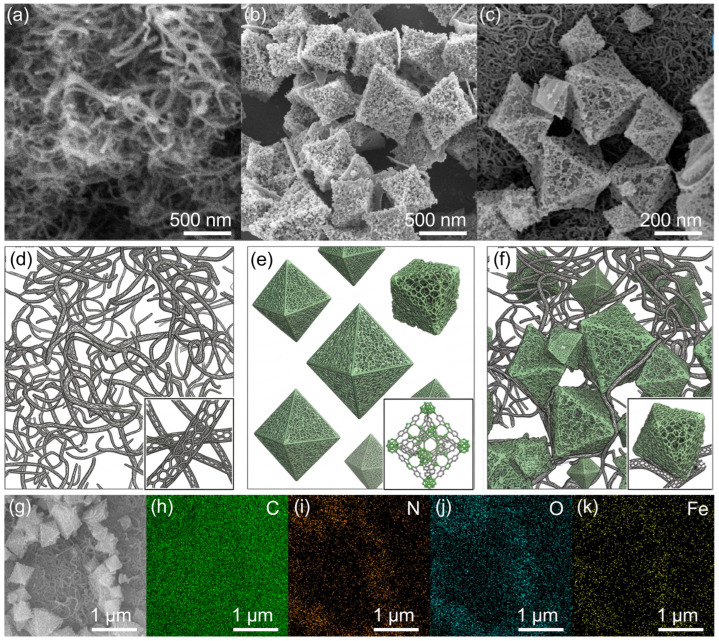
SEM images of (**a**) CNTs, (**b**) NM-101, and (**c**) NM-101/75C. Schematic illustration of (**d**) CNTs, (**e**) NM-101, and (**f**) NM-101/75C. (**g**) SEM image of NM-101/75C. Corresponding elemental mapping images of (**h**) C, (**i**) N, (**j**) O, and (**k**) Fe, respectively.

The carbon nanotubes exhibit a characteristic curved and flexible morphology (Figure 1a,d), intertwining to establish a complex three-dimensional (3D) interconnected porous network. The NM-101 nanocrystals exhibit highly regular octahedral geometries (Figure 1b,e), indicating a high degree of crystallinity. The NM-101/75C composite (Figure 1c,f) exhibits a co-existence of octahedral MOF nanocrystals and CNTs. EDS elemental mapping (Figure 1g–k) confirms a uniform distribution of carbon (C) across the entire architecture, originating from both the CNT scaffolds and the organic linkers. In contrast, nitrogen (N), oxygen (O), and iron (Fe) are predominantly localized within the octahedral domains, consistent with the spatial distribution of the amino-functionalized ligands and oxo-centered iron clusters of the NM-101 framework.

**Figure 2:**
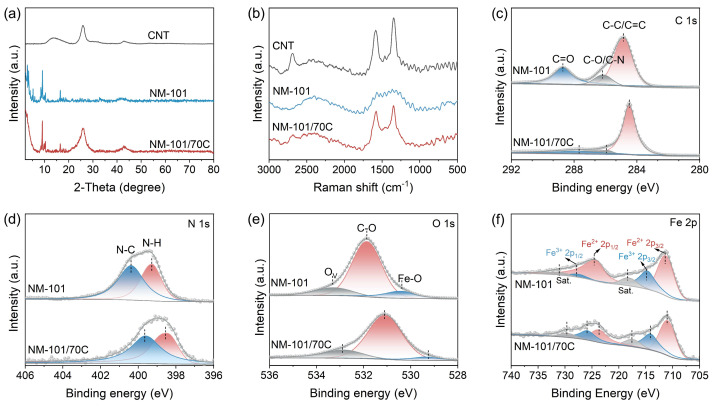
(**a**) XRD patterns and (**b**) Raman spectra of NM-101 and NM-101/XC with varying CNT contents. High-resolution XPS spectra of (**c**) C 1s, (**d**) N 1s, (**e**) O 1s, and (**f**) Fe 2p for the optimized NM-101/75C sample.

The X-ray diffraction (XRD) pattern (Figure 2a) of the NM-101/75C composite displays the characteristic Bragg reflections of MOF-808 superimposed on the broad diffraction features of the carbon nanotubes. The high intensity and sharp profiles of these MOF-related peaks remain preserved even after carbon nanotubes loading. This observation confirms that the structural integrity and excellent crystallinity of the NM-101 framework are rigorously maintained throughout the synthesis of the composite. Raman spectra (Figure 2b) of the NM-101/75C composites predominantly exhibit the characteristic D, G, and 2D bands at 1307, 1586, and 2629 cm^−1^, respectively, which are typical of graphitic carbon frameworks. Notably, the vibrational modes associated with the ligands of the MOF phase are not discernible, suggesting that the structural features of the carbon nanotubes dominate the surface spectral response.

As illustrated in Figure 2c, deconvoluted C 1s XPS spectra of NM-101 exhibit three primary peaks situated at 284.8, 286.2, and 288.7 eV, which are unambiguously assigned to C–C/C=C, C–O/C–N, and C=O functionalities, respectively. As illustrated in Figure 2d, the N 1s spectrum of NM-101 exhibits peaks at 399.3 and 400.4 eV, corresponding to N–H and C–N functionalities, respectively. As shown in the O 1s spectrum of NM-101 (Figure 2e), the peaks centered at 530.5, 531.8, and 533.1 eV are assigned to Fe–O, C–O, and O–H species, respectively. The Fe 2p spectrum of NM-101 exhibits characteristic peaks at 711.3 and 724.5 eV, attributed to the Fe 2p3/2 and Fe 2p1/2 transitions of Fe^2+^, along with peaks at 711.5 and 727.8 eV corresponding to the corresponding states of Fe^3+^ (Figure 2f). Deconvolution of the Fe 2p XPS spectra was performed to estimate the Fe^2+^/Fe^3+^ ratios (Table S1). Compared with pristine NM-101, the NM-101/75C composite exhibits a slightly higher Fe^2+^ proportion, consistent with electron transfer from CNTs to the Fe centers. Such electronic modulation is expected to facilitate charge transfer and optimize the adsorption/desorption of reaction intermediates, thereby enhancing catalytic activity. Nevertheless, the improved performance of NM-101/75C cannot be attributed solely to changes in the Fe^2+^/Fe^3+^ ratio. Instead, it arises from the synergistic effects of CNT-induced electronic interactions with Fe active sites, enhanced electrical conductivity, and improved accessibility of catalytic sites. Therefore, the Fe^2+^/Fe^3+^ ratio serves as an indicator of local electronic structure evolution rather than the sole determinant of catalytic performance. Comparing the composite with the individual NM-101, all characteristic peaks in the C 1s, N 1s, and metal 2p XPS spectra of NM-101/75C exhibit a shift toward lower binding energies. This negative binding energy shift provides strong evidence for an increased electron density at the MOF sites, confirming a directed interfacial electron transfer from the CNTs to the NM-101 framework. Such strong electronic coupling is conducive to accelerating charge-transfer kinetics and modulating the catalytic activity of the composite.

### 3.2. Photothermal Catalytic Performance for Nitronaphthalene Reduction

**Figure 3:**
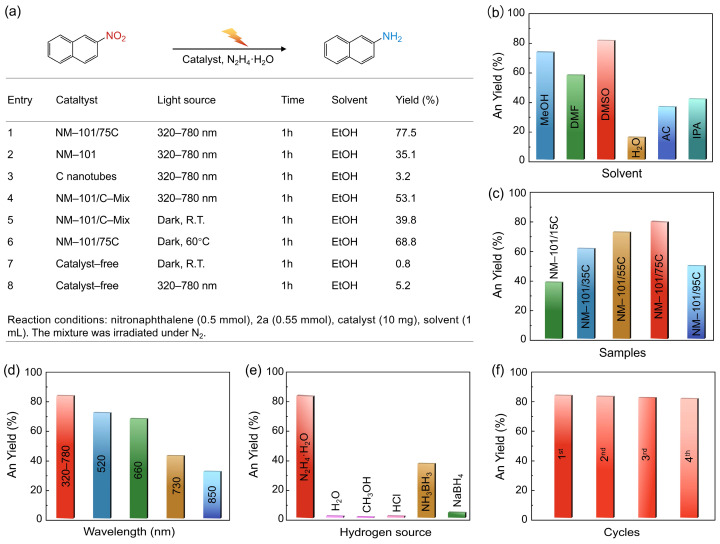
(**a**) Photocatalytic reduction of 1-nitronaphthalene. Optimization of (**b**) solvents, (**c**) catalyst loading, (**d**) wavelengths and (**e**) hydrogen sources. (**f**) Recycling tests.

The photocatalytic reduction performance was evaluated using the hydrogenation of 1-nitronaphthalene to 1-naphthylamine in ethanol for 1 h as a model reaction. Under 320–780 nm irradiation, the NM-101/75C catalyst afforded a yield of 77.5% (Figure 3a, Entry 1), significantly outperforming the pristine NM-101 (35.1%, Entry 2) and CNTs (3.2%, Entry 3). A physical mixture of NM-101 and carbon nanotubes yielded 53.1% (Entry 4), further underscoring the superior performance of the composite. Control experiments in the dark at room temperature resulted in a 39.8% yield (Entry 5), which increased to 68.8% upon heating to 60 °C (Entry 6). Notably, this remains lower than the yield achieved under photothermal conditions, suggesting a synergistic photothermal effect. In the absence of a catalyst, the yields were negligible at 0.8% in the dark (Entry 7) and 5.2% under light irradiation (Entry 8). The influence of solvent on the reaction efficiency was investigated (Figure 3b). Among the tested solvents, DMSO delivered the highest yield (84.4%). MeOH and DMF afforded moderate yields of 77.5% and 58.2%, respectively. IPA and acetone gave lower yields (42.7% and 35.1%), while H_2_O led to a poor yield of 15.2%. Consequently, DMSO was selected as the optimal solvent for subsequent studies. The CNT loading content plays a critical role in determining the optical and catalytic properties of the CNT/NM-101 composites (Figure 3c). As the CNT content increases from 35 to 95 wt%, several competing effects are observed. First, the incorporation of CNTs broadens the light-harvesting range and enhances visible–near-infrared absorption, leading to a gradual decrease in the apparent optical bandgap and improved solar-energy utilization. Second, CNTs serve as conductive pathways that promote charge separation and facilitate interfacial electron transport, thereby suppressing electron–hole recombination. Third, owing to their excellent light-to-heat conversion capability, higher CNT loadings enhance the photothermal effect and increase the local reaction temperature under illumination. At lower CNT contents (35 and 55 wt%), the conductive network and photothermal contribution are not fully developed, limiting the synergistic interaction between CNTs and NM-101. Increasing the CNT loading to 75 wt% significantly improves light absorption, charge transport, and photothermal conversion while maintaining sufficient exposure of the Fe active sites, resulting in the highest catalytic activity. Further increasing the CNT content to 95 wt% does not lead to additional performance enhancement, likely because excessive CNT coverage partially reduces the accessibility of active MOF sites and decreases the relative amount of catalytically active NM-101. Therefore, the superior performance of NM-101/75C arises from an optimal balance between enhanced light harvesting, efficient charge transfer, strong photothermal conversion, and adequate exposure of active sites. To elucidate the wavelength-dependent catalytic activity, the performance of NM-101/75C was evaluated under both broadband and monochromatic irradiation. As illustrated in Figure 3d, the catalyst exhibited significantly enhanced activity under broadband light (320–780 nm) compared to its performance under discrete monochromatic sources at 520, 660, 730, and 850 nm. This superior broadband activity is attributed to the synergistic excitation of multiple electronic transitions within the NM-101/75C heterostructure, allowing for more efficient utilization of the solar spectrum. Control experiments using pristine CNT under identical irradiation conditions (320–780 nm, 520, 660, 730, and 850 nm) revealed only limited catalytic activity across all tested wavelengths, including the NIR region (Figure S4). Although CNTs efficiently absorb light and generate heat through photothermal conversion, their catalytic yields were significantly lower than those of CNT/NM-101, indicating that photothermal heating alone cannot account for the enhanced performance. The superior activity of CNT/NM-101 therefore arises from the synergistic interaction between CNTs and NM-101, where CNTs promote light harvesting, charge transport, and photothermal conversion, while NM-101 provides the catalytically active Fe sites. The intimate CNT-MOF interface enables efficient coupling of these functions, particularly under long-wavelength irradiation. The wavelength-dependent catalytic performance of CNT/NM-101 is governed by the synergistic interplay between photocatalytic and photothermal processes. Under short-wavelength irradiation, catalytic activity is dominated by photoinduced charge generation and transfer within the MOF. As the wavelength shifts toward the red and near-infrared regions, the efficiency of direct photoexcitation in NM-101 decreases, while CNTs continue to harvest light effectively and convert it into heat through nonradiative relaxation. The resulting local temperature increase accelerates reaction kinetics and sustains catalytic activity even under long-wavelength irradiation [47,48,49]. The measurable activity observed at 850 nm further highlights the contribution of the photothermal effect, given that the photon energy at this wavelength is insufficient to efficiently excite NM-101. These results demonstrate that the enhanced performance of CNT/NM-101 originates from the combined effects of photoinduced charge transfer at Fe active sites and CNT-mediated photothermal heating, enabling efficient catalytic conversion across a broad spectral range. As the wavelength increases beyond 700 nm, direct photoexcitation of NM-101 becomes less efficient, and the reaction is increasingly driven by the photothermal contribution of CNTs. Nevertheless, the catalytic activity under near-infrared irradiation does not exceed that under visible light because photocatalytic charge generation is substantially reduced at longer wavelengths. Therefore, the superior performance of NM-101/C originates from the synergistic coupling of photoinduced charge transfer and CNT-mediated photothermal heating rather than from photothermal effects alone. The effect of different hydrogen sources on the reaction yield was examined (Figure 3d). Hydrazine monohydrate (N_2_H_4_·H_2_O) proved to be the most effective, affording the desired product in 84.4% yield. The product formation rate is higher than that of most reported catalysts. In contrast, NH_3_BH_3_ and NaBH_4_ gave significantly lower yields of 41.1% and 4.3%, respectively. Using H_2_O or CH_3_OH or HCl resulted in only trace amounts. Consequently, N_2_H_4_·H_2_O was selected as the optimal hydrogen source for further studies. As depicted in Figure 3f, no significant loss in catalytic activity was observed over four consecutive cycles, indicating excellent stability. The structural stability of NM-101/75C after repeated catalytic cycles was evaluated by XRD and SEM analyses. As shown in Figure S2, the XRD pattern of the recovered catalyst closely resembles that of the fresh sample, with no discernible peak shifts or loss of characteristic diffraction peaks, confirming the preservation of the NM-101 crystalline framework. Furthermore, SEM images (Figure S3) show that the composite retains its original morphology without evident aggregation or structural degradation. The maintained interfacial contact between CNTs and NM-101 further indicates the stability of the NM-101/75C heterostructure during the catalytic process.

### 3.3. Evaluation of Photoelectrochemical Performances

**Figure 4:**
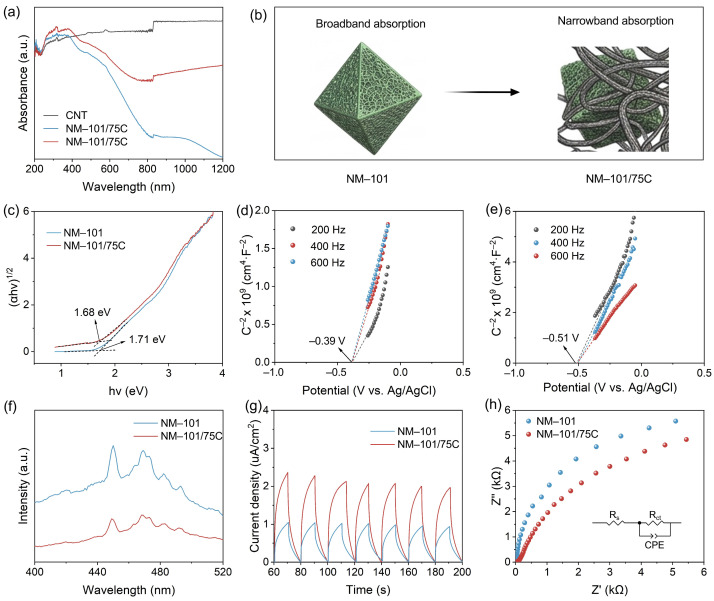
(**a**) UV-vis DRS spectra and (**c**) Kubelka–Munk transformed reflectance spectra of CNTs, NM-101, and NM-101/75C. (**b**) Schematic illustration of optical absorption. Mott-Schottky plots of (**d**) NM-101 and (**e**) NM-101/75C at different frequencies. (**f**) Steady-state PL spectra, (**g**) transient photocurrent responses, and (**h**) EIS Nyquist plots of NM-101 and NM-101/75C.

As illustrated in Figure 4a, the NM-101/75C composite exhibits significantly enhanced light harvesting and a broadened absorption range compared to pristine NM-101, which is attributed to the characteristic full-spectrum absorption of carbon nanotubes. Based on the Kubelka–Munk function, the optical bandgaps (Eg) of NM-101 and NM-101/75C were determined to be 1.71 eV and 1.68 eV, respectively (Figure 4c). The positive slopes in the M-S plots (Figure 4d,e) confirm the n-type semiconductor nature of NM-101 and NM-101/75C. The flat-band potentials (E_fb_) for NM-101 and NM-101/75C were measured at −0.32 V and −0.53 V (vs. Ag/AgCl), respectively. Assuming that the conduction band potential (CB) of an n-type semiconductor is approximately 0.2 V more negative than its flat-band potential, the CB values for NM-101 and NM-101/75C were calculated to be −0.02 V and −0.23 V (vs. NHE). By incorporating the previously determined Eg values, the corresponding valence band potentials (VB) were calculated as 1.69 V and 1.45 V, respectively. The resulting band edge alignments are schematically summarized in Figure 4b.

The steady-state PL spectra (Figure 4f) exhibit a prominent emission peak centered at approximately 450 nm. A marked quenching of the PL intensity is observed for the NM-101/75C composite, indicating a suppressed recombination rate of photogenerated electrons and holes. As shown in Figure 4g, the transient photocurrent response reveals that NM-101/75C exhibits the highest photocurrent density, which signifies superior efficiency in both the generation and separation of photoinduced charge carriers. The EIS Nyquist plots (Figure 4h) demonstrate a significantly smaller arc radius for NM-101/75C compared to pristine NM-101, confirming a substantial reduction in interfacial charge-transfer resistance and faster charge migration. Collectively, those results demonstrate that the integration of NM-101 with carbon nanotubes facilitates effective charge transfer and separation while inhibiting carrier recombination, which serves as the fundamental mechanism for the significantly enhanced photocatalytic activity.

### 3.4. Photothermal Behaviors and Efficiency Evaluation

**Figure 5:**
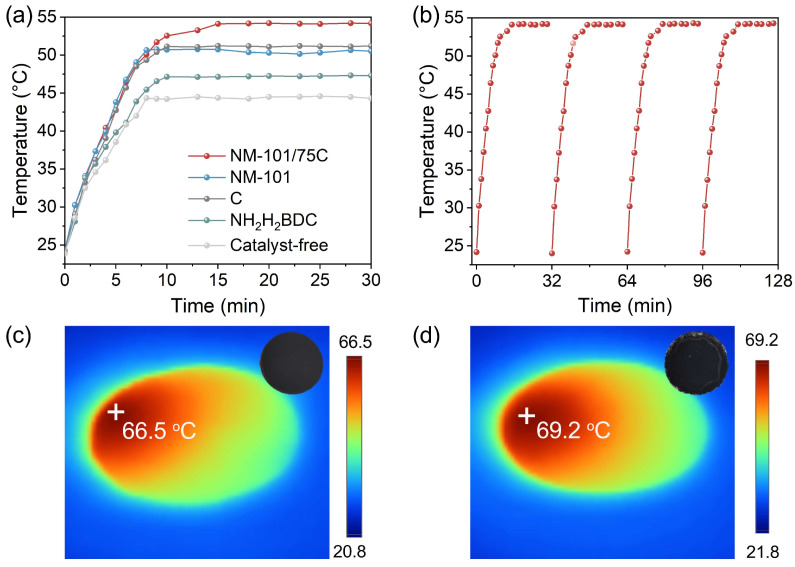
(**a**) Temperature–time curves of different catalysts. (**b**) Cycling temperature–time curves of NM-101/75C. Infrared thermal images of (**c**) NM-101 and (**d**) NM-101/75C; the inset in the upper-right corner of (**c**,**d**) are optical photographs of the NM-101 and NM-101/75C samples, respectively.

To quantify the photothermal performance of the materials, the temperature evolution of the reaction systems was monitored using a thermocouple detector. As illustrated in the temperature–time profiles (Figure 5a), the NM-101/75C system exhibited a significantly higher steady-state temperature compared to other catalysts, reaching approximately 54 °C. This photothermal effect remained remarkably consistent over four consecutive cycles, with the peak temperature maintained at about 54 °C (Figure 5b), thereby confirming the long-term photostability and robust thermal generation of the composite. The photothermal conversion efficiency (η) of the NM-101/75C composite was calculated to be 62.2%. Furthermore, infrared (IR) thermal imaging (Figure 5c) under 660 nm light irradiation revealed that the surface temperature of NM-101/75C (69.2 °C) exceeded those of the pristine NM-101 (65.5 °C) samples (Figure 5d). Collectively, these results demonstrate that the integration of carbon nanotubes significantly enhances the photothermal conversion efficiency and thermal management capabilities of the NM-101 framework under light excitation.

### 3.5. Photocatalytic Mechanism Studies

**Figure 6:**
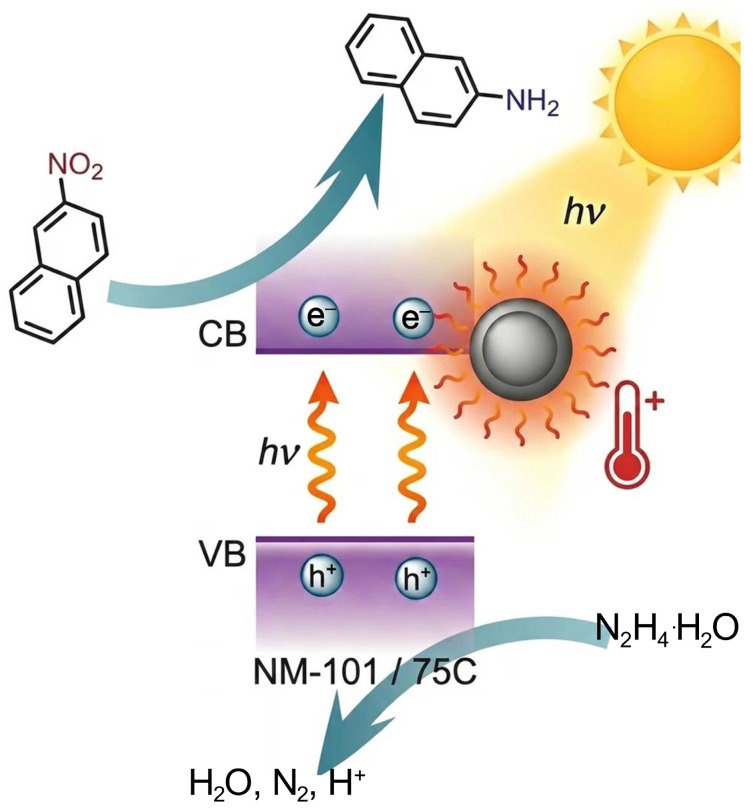
Schematic illustration of the proposed mechanism for the reduction in nitronaphthalenes over the NM-101/C composite.

Integrating the spectroscopic and electrochemical results, a plausible photothermal catalytic mechanism is proposed in Figure 6. In this NM-101/CNT composite, the CNTs function not only as a conductive scaffold but also as a potent photothermal antenna. The CNTs leverage full-spectrum solar harvesting to drive localized photothermal effects, which overcomes kinetic barriers, thereby accelerating surface reaction kinetics. Simultaneously, photoinduced electrons migrate from the VB to the CB of NM-101. The synergistic effect of the thermally-activated surface and the enriched electron density from the CNT reservoir markedly bolsters the thermodynamic driving force for reduction. These high-energy electrons then reduce 1-nitronaphthalene to 1-naphthylamine via a thermally-assisted proton-coupled electron transfer (PCET) process. Concurrently, the photogenerated holes in the VB facilitate the sacrificial oxidation of hydrazine hydrate (N_2_H_4_·H_2_O) into N_2_, H_2_O and H^+^. This spatial charge separation, coupled with the localized photothermal effect mediated by the CNT network, is identified as the pivotal factor behind the enhanced catalytic performance.

## 4. Conclusions

In conclusion, we have successfully developed a robust and scalable solvothermal strategy for the rational construction of an advanced NH_2_-MIL-101(Fe)/carbon nanotube (NM-101/C) composite as a highly efficient and sustainable photothermal catalyst. The optimized NM-101/75C composite exhibits exceptional catalytic performance and selectivity toward the reduction in nitronaphthalenes under NIR light irradiation, achieving an outstanding yield of 84.4% within 1 h, which significantly outperforms the individual benchmarks. Systematic control experiments and spectroscopic investigations verify that the superior activity originates from a powerful synergistic effect at the MOF-CNT interface. Specifically, the integrated CNTs fulfill a dual-functional role by simultaneously enhancing light harvesting and accelerating charge carrier separation, while the high photothermal conversion efficiency ensures rapid reaction kinetics. This work not only offers a viable approach to designing high-performance photothermal catalysts but also provides critical, generalizable insights into interface engineering of MOF-based materials for sustainable chemical manufacturing.

## Data Availability

The original contributions presented in this study are included in the article/Supplementary Material. Further inquiries can be directed to the corresponding authors.

## Supplementary Materials

The following supporting information can be downloaded at: https://www.mdpi.com/article/10.3390/nano16130817/s1, Catalyst characterization; Photocatalytic H_2_O_2_ production. Figure S1. Schematic diagram of the band structure. Figure S2. XRD of NM-101/75C before and after reaction. Figure S3. SEM of NM-101/75C after reaction. Figure S4. Dependence of aniline yield on wavelength. Table S1. Quantitative analysis of Fe species from high-resolution Fe 2p XPS spectra. Table S2. Comparison of reported catalysts for the reduction of 1-nitronaphthalene.
