# Supplementary material for: Boosting Photo-to-Thermal Conversion and 1-Nitronaphthalene Reduction in Fe-MOF via Incorporating Carbon Nanotubes Heat-Storage Cocatalyst
Source: Nanomaterials (Basel). 2026 Jul 2;16(13):817. doi: 10.3390/nano16130817 (PMC13363670; doi:10.3390/nano16130817)
Supplement: Supplementary file 1 [file nanomaterials-16-00817-s001.zip › nanomaterials-4362784-supplementary.pdf]

Boosting photo-to-thermal conversion and 1-nitronaphthalene reduction of Fe-MOF via incorporating carbon nanotubes heat-storage cocatalyst

Ying-Cong Wei [1], Zhuang Miao [1], Zhipeng Xie [2*] and Xiong-Feng Ma [1*]

[1] College of Engineering, Xi'an International University, Xi'an 710077, China

[2] Department of Chemistry, The Chinese University of Hong Kong, New Territories, Hong Kong 999077, China

* Correspondence: zpxie@hku.hk (Z. Xie), maxiongfeng1992@163.com (X. Ma)

1. Materials and Characterization

All chemicals and reagents were obtained from suppliers and were directly used without further purification. 2-Aminoterephthalic acid and $FeCl_3.6H_2O$ were purchased from Adamas. N, N-dimethylformamide (DMF, AR) was bought from Xilong Scientific.

Powder X-ray diffraction (PXRD) patterns were collected using a Bruker D8 ADVANCE diffractometer with Cu Kα radiation (λ = 1.54056 Å). Scanning electron microscopy (SEM) was conducted on a Hitachi SU-8010 instrument. Transmission electron microscopy (TEM), high-angle annular dark-field scanning transmission electron microscopy (HAADF-STEM), and energy-dispersive X-ray (EDX) elemental mapping were executed using an FEI Talos F200X microscope operating at 200 kV. X-ray photoelectron spectroscopy (XPS) was performed on a PHI 5000 VersaProbe III spectrometer using an Al Kα (1486.6 eV) achromatic source, with all binding energies calibrated to the C 1s peak at 284.8 eV. UV-vis diffuse reflectance spectra (UV-vis DRS) were recorded from a Shimadzu UV-3600 Plus spectrophotometer utilizing $BaSO_4$ as the reference. Raman spectra were acquired with an Ocean Optics QE Pro-Raman spectrometer equipped with a 785 nm excitation laser).

2. Photoelectrochemical (PEC) measurements

Transient photocurrent responses, electrochemical impedance spectroscopy (EIS), and Mott-Schottky plots were performed on a CHI 660E electrochemical workstation (Chenhua) employing a conventional three-electrode configuration. This setup comprised a Pt foil as the counter electrode and a saturated Ag/AgCl as the reference electrode. To fabricate the working electrode, a homogeneous slurry was first prepared by dispersing the photocatalyst (5 mg) in a mixture of isopropanol (i-PrOH, 0.9 mL) and Nafion (0.1 mL). Subsequently, 100 μL of this slurry was drop-cast onto an indium tin oxide (ITO) conductive glass substrate (active area: 1 cm^2), followed by drying in a vacuum oven at 80 °C. An acetonitrile solution containing 0.1 M tetrabutylammonium hexafluorophosphate served as the electrolyte.

3. Photothermal conversion efficiency

The photothermal conversion efficiency (η) was calculated using the equation:

$$\eta = \frac{hS \cdot \Delta T_{max} - Q_{dis}}{P \cdot (1 - 10^{-A})}$$

where P is the actual output power of the 808 nm laser; A is the absorbance of the sample at 808 nm; ΔT_{max} is the maximum temperature rise of the sample solution at the steady state under laser irradiation; Q_{dis} is the background photothermal effect of the pure solvent under identical laser conditions, used to subtract the thermal contribution from the container and solvent; and hS is the total heat transfer coefficient (W/K) of the system.

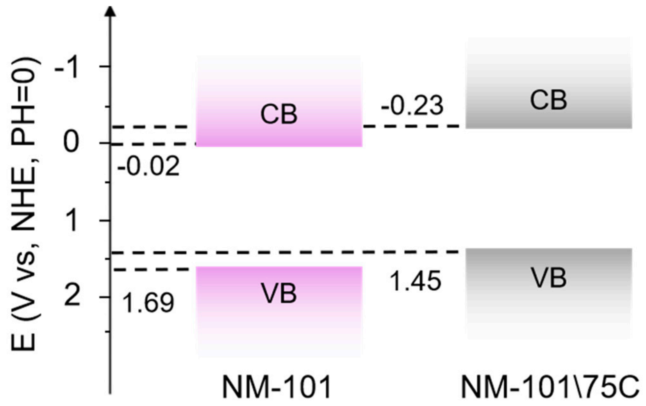

Figure S1. Schematic diagram of the band structure.

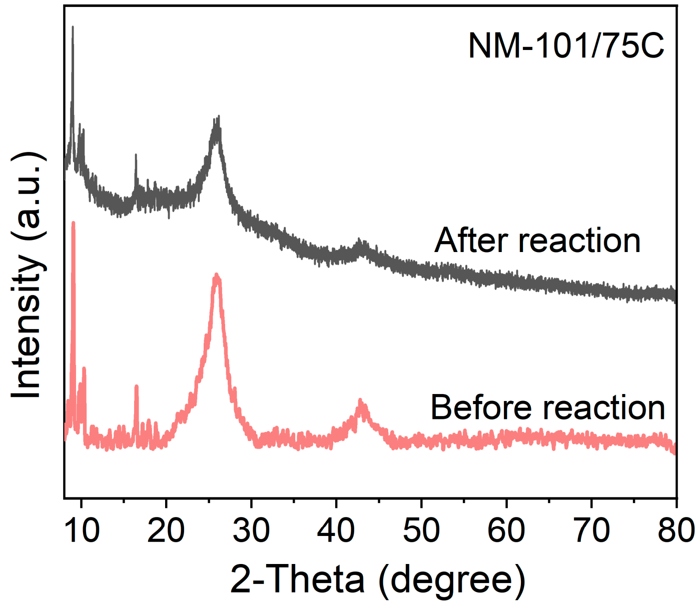

Figure S2. XRD of NM-101/75C before and after reaction.

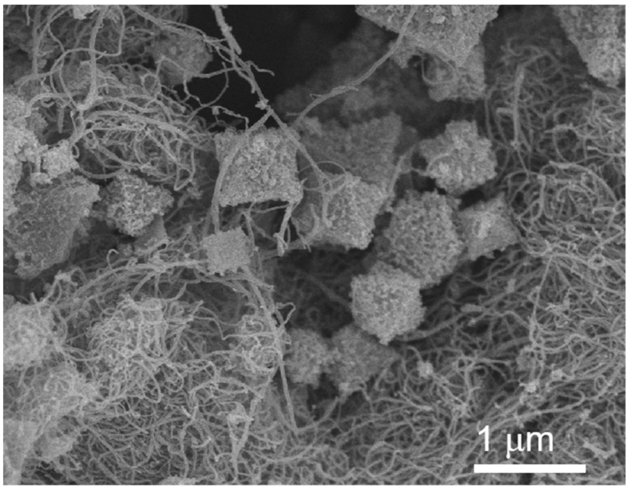

Figure S3. SEM of NM-101/75C after reaction.

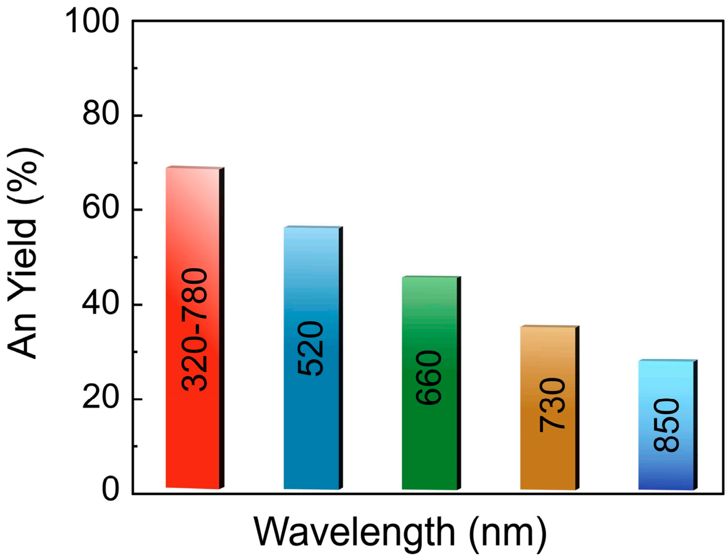

Figure S4. Dependence of aniline yield on wavelength.

Table S1. Quantitative analysis of Fe species from high-resolution Fe 2p XPS spectra.

Sample	Fe^{2+} fraction (%)	Fe^{3+} fraction (%)	Fe^{2+}/Fe^{3+} ratio
NM-101	32.8	67.2	0.49
NM-101/75C	36.7	63.3	0.58

Table S2. Comparison of reported catalysts for the reduction of 1-nitronaphthalene.

Sample	Time (min)	Yield (%)	Ref.
TBPB	120	75.4	[1]
Co_xNi_y@NC/SiO_2	330	100	[2]
Ni-Zn/AC-350	300	96.8	[3]
NM-101/75C	60	84.4	This work

[1] Ujjal, M.; Sen, S.; Singhand, G. Advances in hydrogen sulphide utilisation: phase transfer catalysed selective reduction of nitronaphthalene. *RSC Adv.* **2015**, *5*, 102942-102952.

[2] Lan, X.; Zhong, M.; Dai, W.; Liu P. High-Performance Co_xNi_y@NC/SiO_2 Catalysts Derived from ZIF-67 for Enhanced Hydrogenation of 1-Nitronaphthalene. *Catalysts* **2026**, *16*, 93.

[3] Lei, H.; Lv, Y.; Wu, S.; Liu, P.; Xiong, W.; Hao, F.; Luo, H. Activated carbon supported bimetallic catalysts with combined catalytic effects for aromatic nitro compounds hydrogenation under mild conditions. *Appl. Catal. A-Gen.* **2019**, *577*, 76-85.
